# Minor differences in body condition and immune status between avian influenza virus-infected and noninfected mallards: a sign of coevolution?

**DOI:** 10.1002/ece3.1359

**Published:** 2014-12-31

**Authors:** Jacintha G B van Dijk, Ron A M Fouchier, Marcel Klaassen, Kevin D Matson

**Affiliations:** 1 Department of Animal Ecology, Netherlands Institute of Ecology (NIOO-KNAW)Droevendaalsesteeg 10, 6708 PB, Wageningen, The Netherlands; 2 Department of Viroscience, Erasmus MCPO Box 2040, 3000 CA, Rotterdam, The Netherlands; 3 Centre for Integrative Ecology, School of Life and Environmental Sciences, Deakin UniversityLocked Bag 20000, Geelong, Victoria, 3220, Australia; 4 Animal Ecology Group, Centre for Ecological and Evolutionary Studies, University of GroningenPO Box 11103, 9700 CC, Groningen, The Netherlands; 5 Resource Ecology Group, Wageningen University, Droevendaalsesteeg 3a6708, PB Wageningen, The Netherlands

**Keywords:** *Anas platyrhynchos*, infectious disease, body mass, migrant, pathogen, shedding, wild birds

## Abstract

Wildlife pathogens can alter host fitness. Low pathogenic avian influenza virus (LPAIV) infection is thought to have negligible impacts on wild birds; however, effects of infection in free-living birds are largely unstudied. We investigated the extent to which LPAIV infection and shedding were associated with body condition and immune status in free-living mallards (*Anas platyrhynchos*), a partially migratory key LPAIV host species. We sampled mallards throughout the species' annual autumn LPAIV infection peak, and we classified individuals according to age, sex, and migratory strategy (based on stable hydrogen isotope analysis) when analyzing data on body mass and five indices of immune status. Body mass was similar for LPAIV-infected and noninfected birds. The degree of virus shedding from the cloaca and oropharynx was not associated with body mass. LPAIV infection and shedding were not associated with natural antibody (NAbs) and complement titers (first lines of defense against infections), concentrations of the acute phase protein haptoglobin (Hp), ratios of heterophils to lymphocytes (H:L ratio), and avian influenza virus (AIV)-specific antibody concentrations. NAbs titers were higher in LPAIV-infected males and local (i.e., short distance) migrants than in infected females and distant (i.e., long distance) migrants. Hp concentrations were higher in LPAIV-infected juveniles and females compared to infected adults and males. NAbs, complement, and Hp levels were lower in LPAIV-infected mallards in early autumn. Our study demonstrates weak associations between infection with and shedding of LPAIV and the body condition and immune status of free-living mallards. These results may support the role of mallards as asymptomatic carriers of LPAIV and raise questions about possible coevolution between virus and host.

## Introduction

Wildlife pathogens can alter host fitness, for instance, by affecting an animal's ability to grow (Burthe et al. 2008), reproduce (de Crespigny and Wedell 2006), or survive (Burthe et al. 2008; Mayack and Naug 2009). Disease outbreaks can have detrimental effects on population numbers, as exemplified by the historic outbreaks of rinderpest in African ungulates at the end of the 20th century (Plowright 1982), and canine distemper in lions and seals in the late 1990s and early 2000 (Roelke-Parker et al. 1996; Kuiken et al. 2006). Yet pathogens can also have more subtle effects which may impair host fitness, such as reduced foraging or decreased activity (Bradley and Altizer 2005; Venesky et al. 2009). Susceptibility to pathogen infection may differ between hosts. For example, West Nile virus negatively impacted populations of North American corvids, while other passerines and members of other orders apparently tolerated infection without significant morbidity (LaDeau et al. 2007). Whether interspecific differences in the effects of pathogens result from different degrees of coevolution (i.e., go through a process of reciprocal, adaptive genetic changes; Woolhouse et al. 2002) is poorly understood. A well-known example of coevolution is the European rabbit–myxoma virus system, where phenotypic changes were observed in both pathogen and host after introduction of the virus into a naïve rabbit population (Fenner and Fantini 1999). Studying effects of pathogens on wildlife are useful for understanding the impacts on host fitness, potential consequences for populations, and, more generally, the role of coevolution.

A common pathogen that circulates naturally in wild birds is low pathogenic avian influenza virus (LPAIV). This virus predominantly infects birds inhabiting wetlands and aquatic environments (orders *Anseriformes* and *Charadriiformes*), which are considered the major natural LPAIV reservoir (Webster et al. 1992). Experimental infection studies under laboratory conditions show that LPAIV causes only mild disease in these species (for review see Kuiken 2013). However, there are only few studies on the effects of LPAIV infection in free-living waterfowl. Studies on Bewick's swans (*Cygnus columbianus bewickii*) and mallard ducks (*Anas platyrhynchos*) showed that individuals that were naturally infected with LPAIV had a lower body mass than noninfected individuals (van Gils et al. 2007; Latorre-Margalef et al. 2009a). This result was also observed in naturally LPAIV infected greater white-fronted geese (*Anser albifrons albifrons*), although in specific years only (Kleijn et al. 2010). As these studies involved free-living birds, cause-and-effect relationships between LPAIV infection and body mass are difficult to assess (Flint and Franson 2009; Latorre-Margalef et al. 2009b). This also applies when linking LPAIV infection to movement and migration of free-living birds. Mallards that were naturally infected with LPAIV had lower regional movements than noninfected individuals (J. G. B. van Dijk, unpublished data). LPAIV-infected Bewick's swans, besides feeding at reduced rates, delayed their migration with a month (van Gils et al. 2007). However, sample size in the latter study was low, and similar studies on the same or related species yielded inconclusive results (Latorre-Margalef et al. 2009a; Hoye 2011). These studies, conducted in few waterfowl species that vary in LPAIV susceptibility, highlight our limited understanding of effects of LPAIV infection on host fitness.

Studying LPAIV infections in free-living waterfowl is of significant socio-economic importance, because LPAIV subtypes H5 and H7 may become highly pathogenic (HPAIV) after introduction into poultry. HPAIV is lethal for poultry and may cause illness and occasional deaths in humans and wild birds (Alexander 2007). HPAIV H5N1, a notorious subtype that emerged in Asia in 1996, has led to the culling of hundreds of millions of poultry and to almost 400 human deaths (World Health Organization 2014). Migratory waterfowl are frequently suggested to be involved, at least partly, in the global spread of HPAIV H5N1 (Kilpatrick et al. 2006; Si et al. 2009), assuming infection does not alter their movement abilities (Gaidet et al. 2010). Although experimental infection studies of HPAIV H5N1 show that specific host species, such as mallards, may abundantly excrete virus without clinical or pathologic signs of disease (Keawcharoen et al. 2008), free-living waterfowl have not been conclusively implicated in the global spread of HPAIV. The urgency of the question of whether free-living waterfowl can indeed serve as asymptomatic carriers of LPAIV increased recently with the outbreak of LPAIV H7N9 in China. This disease can be pathogenic for humans, while wild birds and domestic poultry show no apparent clinical symptoms (Kreijtz et al. 2013). Therefore, studying effects of LPAIV infection in free-living waterfowl is critical to understand the role of these birds as potential carriers of this infectious disease.

Dabbling ducks of the *Anas* genus, and particularly mallards, are frequently infected with LPAIV (Olsen et al. 2006). Mallards are known to be infected with almost all LPAIV subtypes that have been found in birds to date (H1–H16, N1–N9; Kawaoka et al. 1990; Röhm et al. 1996; Olsen et al. 2006). Experimentally infected mallards shed high LPAIV titers (Kuiken 2013), briefly increase their body temperature by 0.5°C at the start of virus shedding (Jourdain et al. 2010), but otherwise exhibit negligible signs of disease (Kuiken 2013). Additionally, mallards mount two types of antibody responses, each with a distinctive time course. A highly specific antibody response, which is relatively short lived (i.e., up to a few weeks; Kida et al. 1980), is mounted against the infecting LPAIV subtype. A LPAIV subtype nonspecific response by antinucleoprotein (anti-NP) antibodies (i.e., antibodies that bind to highly conserved nucleoprotein epitopes on LPAIV particles) is longer lasting, with antibodies present for 6–15 months (Fereidouni et al. 2010). It remains unclear how free-living mallards cope with natural LPAIV infections and whether their energetic and immunological statuses are impacted.

**Figure 1:**
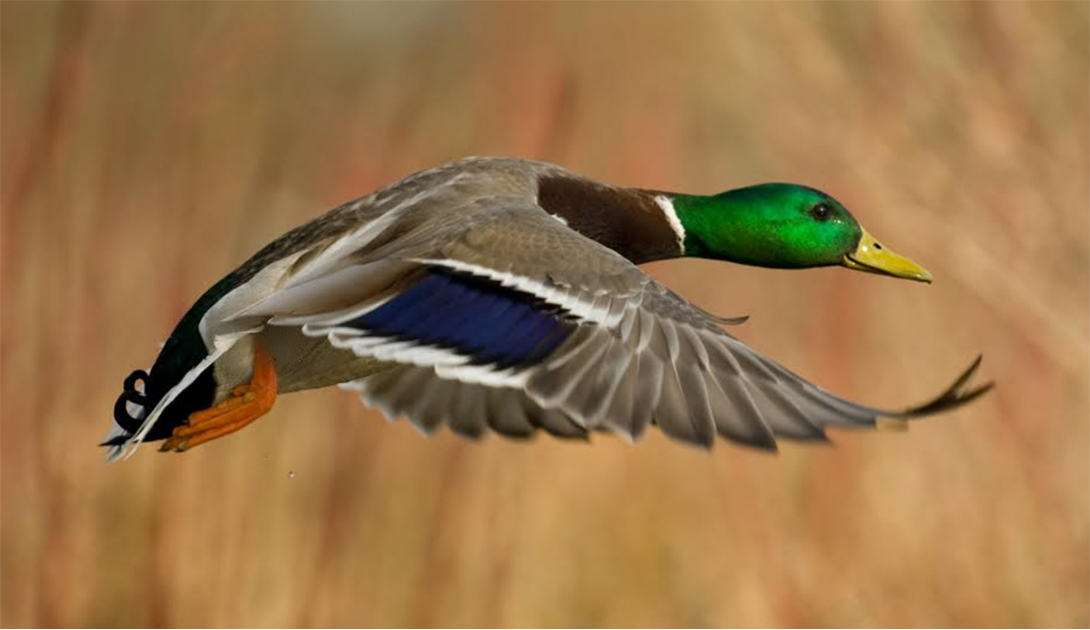
Male mallard (*Anas platyrhynchos*; picture taken by D.J. Brown).

The aim of our study was to investigate the extent to which LPAIV infection and shedding were associated with body condition and immune status in free-living mallards (Fig.1). We comprehensively sampled mallards on their wintering grounds during the autumn LPAIV infection peak. Autumn is generally the period that LPAIV infection in mallard populations is the highest in the northern hemisphere (van Dijk et al. 2014a; Latorre-Margalef et al. 2014). In our investigation of the interactions between body condition and immune status, we considered effects of bird age, sex, and migratory strategy. Our study population consists of both migratory and resident birds (throughout Europe mallards are partially migratory; Scott and Rose 1996). Effects of LPAIV infection in juveniles may be more profound than in adults, because juveniles are immunologically naïve and immunity to LPAIV is likely acquired with age (Munster et al. 2007; Latorre-Margalef et al. 2009a). Due to sex differences in body condition, immune status, and physiology in general, effects of LPAIV infection may differ between males and females (Zuk and McKean 1996). Males are less likely to have anti-NP antibodies than females, although LPAIV infection and shedding are similar between the sexes (Munster et al. 2007; van Dijk et al. 2014a). Effects of LPAIV infection may also differ between migratory and resident birds, because energetic demands of migration may compromise immune function and nutritional status in migratory birds (Owen and Moore 2006). Indeed in autumn, migratory mallards were more frequently infected with LPAIV than residents, although virus shedding was similar, and migrants had low anti-NP antibodies (van Dijk et al. 2014a).

## Materials and Methods

### Sampling

From August until December 2010, coinciding with the major annual LPAIV infection peak, mallards were caught using swim-in traps (i.e., a duck decoy; Payne-Gallwey 1886) located near Oud Alblas (51°52′38″N, 4°43′26″E) in the Alblasserwaard, the Netherlands. On average, we visited the decoy six times per month (ca. 5 days in between catches) and captured approximately nine individuals per visit. Each individual was marked with a metal ring and categorized based on plumage characteristics as male or female and as juvenile (<1 year) or adult (>1 year; Boyd et al. 1975). We measured tarsus length (nearest 0.01 mm; Byers and Cary 1991), head + bill length (nearest 0.1 mm), and wing length (maximum wing chord, nearest 1 mm; Baker 1993). A digital balance was used to measure body mass (nearest 1 g; Kern EMB-2200-0). We used sterile cotton applicators to swab the cloaca and the oropharynx, as experimental infection studies in mallards show that LPAIV replicates in the intestinal tract and, in lower titers, in the respiratory tract (Kida et al. 1980). Swabs were stored individually in transport medium (Hank's balanced salt solution with supplements; Munster et al. 2009) at 4°C and transported to Erasmus MC for analysis within 7 days of collection (Munster et al. 2009). We collected blood samples (<1 mL and <2% of the circulating blood volume) from the brachial vein and used small aliquots (several drops) to make smears for leukocyte enumeration. The remainder was allowed to clot for approximately 6 h before centrifugation to separate serum and cell fractions (Hoye 2012). Serum samples were stored at −20°C for several months until analysis. We collected the tip (1–2 cm) of the first primary feather (P1) of the right wing for stable hydrogen isotope analysis. Feather samples were stored at room temperature in sealed plastic bags. We secured approval from the Animal Experiment Committee of Erasmus MC (protocol 122-10-20) and the Dutch Ministry of Economic Affairs (Flora and Fauna permit FF/75A/2009/067).

### Virus detection

Cloacal and oropharyngeal swabs were analyzed separately for the presence of influenza A virus. For full details on RNA isolation and virus detection, see Munster et al. (2009). In short, RNA was isolated using a MagNA Pure LC System (no. 12236931001) with the MagNA Pure LC Total Nucleic Acid Isolation Kit (no. 0303850500; Roche Diagnostics, Almere, the Netherlands). Influenza A virus was detected using a generic real-time reverse transcriptase PCR assay targeting the matrix gene (M RRT-PCR). Amplification and detection were performed on an ABI 7700 machine with a TaqMan EZ RT-PCR core reagents kit (no. N808-0236; Applied Biosystems, Nieuwerkerk aan den IJssel, the Netherlands). Samples were considered positive for influenza A virus if the cycle threshold (*C*_T_) value, which is the first real-time amplification cycle in which matrix gene amplification was detected, was ≤40. The *C*_T_-value is inversely proportional to the number of virus particles in a sample.

### Immune assays

We examined five immunological indices: (1) natural antibodies (NAbs); (2) complement; (3) haptoglobin (Hp); (4) ratio of heterophils to lymphocytes (H:L ratio); and (5) anti-NP antibodies.

A hemolysis–hemagglutination assay with rabbit red blood cells (no. RBA050; HemoStat Laboratories, Dixon, CA) was used to quantify nonspecific NAbs (i.e., agglutination score) and NAb-mediated complement activation (i.e., lysis score) in serum, as described by Matson et al. (2005). All scans of individual samples were randomized and scored blindly. NAbs (predominantly immunoglobulin IgM) and complement are part of the innate immune system and act as a first line of defense against infections (Ochsenbein and Zinkernagel 2000). NAbs are produced in the absence of exogenous antigenic stimulation and are supposedly unaffected by current infection (Ochsenbein and Zinkernagel 2000). Complement is a group of proteins involved in inflammation that can be activated directly by pathogens or indirectly by antigen-bound antibodies (Müller-Eberhard 1988). NAbs and complement titers can be used to characterize innate humoral immunity in wild birds (Matson et al. 2005).

Hp concentrations (mg·mL^−1^) in serum were quantified using a commercially available assay (no. TP-801; Tridelta Development Limited, Maynooth, County Kildare, Ireland), following manufacturer's instructions with several modifications (i.e., a wider range of dilutions in the standard curve, the baseline quantification of serum absorbances at the normal assay wavelength, and the colorimetric quantification of hemolysis in serum samples; Matson et al. 2012a). Hp is an acute phase protein that binds free hemoglobin to prevent it from providing nutrients to pathogens. Concentrations of Hp typically increase in response to acute infection, inflammation, or trauma (Delers et al. 1988). Chickens experimentally inoculated with infectious bronchitis virus show elevated Hp concentrations (Asasi et al. 2013; Seifi et al. 2014).

The first 100 leukocytes per blood smear were classified and enumerated. Smears were evaluated blindly by one veterinary diagnostic laboratory technician (European Veterinary Laboratory, Woerden, the Netherlands). Based on these counts, we calculated the ratio between heterophils and lymphocytes (i.e., H:L). Heterophils and lymphocytes are the two dominant leukocytes that mediate nonspecific immunity and specific antibody responses, respectively (Campbell 1995). An increase in H:L ratio can reflect stress and susceptibility to infection (Davis et al. 2008). H:L is not affected by handling time (Davis 2005).

To measure serum concentrations of anti-NP antibodies, we used a commercially available blocking enzyme-linked immunosorbent assay (no. 99-53101 bELISA MultiS-Screen Avian Influenza Virus Antibody Test Kit; IDEXX Laboratories, Hoofddorp, the Netherlands) following manufacturer's instructions. Samples were tested in duplicate, and each plate contained two positive and two negative controls. The absorbance (i.e., OD value) was measured at 620 nm using an infinite M200 plate reader (Tecan Group Ltd, Männedorf, Switzerland). We used OD values as a relative measure of anti-NP antibody concentration. A recent study validated the use of OD values in mallard sera as a quantitative estimate of anti-NP antibody concentration (van Dijk et al. 2014b).

### Stable isotope analysis

Stable hydrogen isotope ratios (*δ*^2^H) in feathers were used to help assess the molting location of mallards, and thereby their migratory strategy (i.e., migrant or resident). Mallards that breed in Finland, Sweden, the Baltic, and northwest Russia migrate in autumn and winter in the area spanning from Denmark to northern France and Britain. As a result, migratory mallards mix with resident individuals in north-western Europe, including the Netherlands (Scott and Rose 1996).

For full details, see van Dijk et al. (2014c). In short, feathers were cleaned with 2:1 chloroform:methanol solvent mixture to remove surface contaminants and oils and air-dried overnight in a fume hood. Feather samples were placed into silver capsules, folded into tiny balls, and stored in 96-well trays. Trays were shipped to the Colorado Plateau Stable Isotope Laboratory (Northern Arizona University, Flagstaff, AZ). Stable hydrogen isotope analyses were performed there on a Delta Plus XL isotope ratio mass spectrometer equipped with a 1400 C TC/EA pyrolysis furnace. Feather *δ*^2^H are reported in units per mil (‰) relative to the Vienna Standard Mean Ocean Water-Standard Light Antarctic Precipitation (VSMOW-SLAP) scale.

Signatures of stable isotopes in animal tissue, including feathers, are a reflection of local food webs (Peterson and Fry 1987). Precipitated water moves up the food chain and is eventually incorporated into feathers during their growth (Hobson 1999). There is a close correlation between feather *δ*^2^H and amount-weighted growing-season *δ*^2^H in precipitation (Hobson and Wassenaar 1997), the latter of which exhibits a gradient across Europe (Bowen et al. 2005). A similar gradient in *δ*^2^H of European mallard feathers enables assessment of the geographic location where individuals molted their feathers (van Dijk et al. 2014c).

In the study of van Dijk et al. (2014a), mallards sampled during the autumn LPAIV infection peak were classified as (1) resident; (2) local migrant (i.e., short distance); and (3) distant migrant (i.e., long distance) based on feather *δ*^2^H and additional criteria, such as time of capture, recapture rate and whether they were in molt. In this study, we used similar criteria to assess the migratory strategy. Residents were captured during molt, that is, they grew their feathers near the study site, and were recaptured multiple times either before or during our focal period in autumn. Local and distant migrants were captured and sampled only once in autumn.

### Data analysis

The dataset contained individuals that were captured and sampled either once (*n *=* *266) or multiple times (*n *=* *19; Table1). Of the 99 LPAIV-positive samples, 8% of the birds were infected in the cloaca, 57% in the oropharynx, and 35% of the birds were infected in both cloaca and oropharynx. As body mass and the immunological indices did not differ between these three groups of samples (linear models (LMs): all *P *>* *0.05), we considered a bird to be LPAIV-positive when either its cloacal or its oropharyngeal sample tested positive.

**Table 1:** Number of samples collected from primary captures (P) and recaptures (R) of mallards, by age (i.e., juvenile: <1 year, adult: >1 year) and sex from August until December 2010. From all individuals, cloacal and oropharyngeal swabs, sera samples and body mass measurements (*n *=* *287) were collected

Age	Sex	Resident		Local migrant	Distant migrant
		Primary	Recapture		
Juvenile	Male	6	3	26	25
	Female	6	1	8	9
Adult	Male	31	14	30	30
	Female	22	3	44	29
Total		65	21	108	93

Collinearity between all the response variables was tested with Pearson correlation (*r*; Table S1). Although several variables were significantly correlated, in all cases *r*^2^ was weak to moderate and therefore all variables were used separately as response variables to test the association with LPAIV infection and shedding. All response variables, except agglutination scores and anti-NP antibody concentrations, were log10-transformed to meet the assumption of normality. Anti-NP antibody concentrations were minusLN-transformed so that high values indicated high concentrations of AIV antibodies. As an index of body size, we used the first principal component (PC1) of a PC analysis of tarsus, head + bill, and wing lengths. PC1 explained 79% of the variance.

We used R 2.14.1 for all analyses (R Development Core Team 2012). Monthly differences in viral prevalence and the degree of virus shedding from the cloaca and oropharynx were tested using, respectively, generalized linear models (GLM) and LMs, with month as fixed factor. Linear mixed models (LMMs; Package ‘lme4’, Bates et al. 2012) were used to test the association between LPAIV infection and six response variables: body mass, agglutination score, lysis score, Hp concentration, H:L ratio, and anti-NP antibody concentration. The models included LPAIV infection status (yes/no), age (juvenile/adult), sex, migratory strategy (resident, local migrant, distant migrant), and month as fixed factors, as well as four two-way interactions between infection status and age, sex, migratory strategy and month, and individual bird as random factor. Month is included in the LMMs, because body mass and immune status may vary over the year (Hegemann et al. 2012). The interaction between LPAIV infection and month is included in the models as this could partly incorporate the difference between mainly primary infections early in autumn and secondary infections late in autumn. LMs were used to test the associations between the degree of virus shedding (*C*_T_-value) from the cloaca and oropharynx and the six response variables. Separate LMs were performed for cloacal and oropharyngeal samples. *C*_T_-value, age, sex, migratory strategy, month, and all two-way interactions with *C*_T_-value were included in the LMs. The fixed factors age, sex, migratory strategy, and month were merely included in the models to conduct the interactions. All models of body mass included PC1 to correct for bird size, and models of Hp concentration included sample redness to correct for hemolysis, which can affect the Hp assay (Matson et al. 2012a). Tukey's post hoc tests were performed to detect differences among different migratory strategies and months. The significance level (*α*) equaled 0.05. With the log-transformed variables, the reported mean values and SE were back-transformed.

## Results

**Figure 2:**
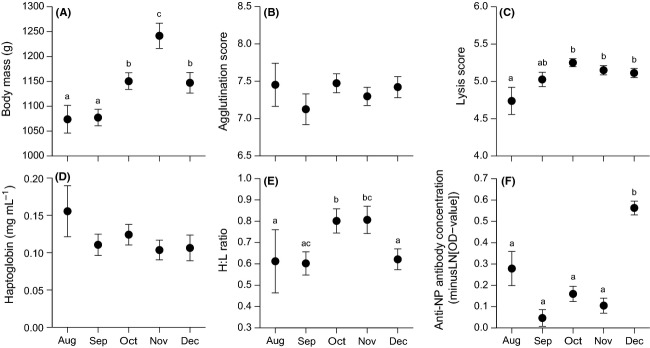
Raw data of body mass and immune status (mean ± SE) of free-living mallards from August until December 2010. (A) Body mass, (B) agglutination score (NAbs), (C) lysis score (complement), (D) haptoglobin (Hp) concentration, (E) H:L ratio (heterophils to lymphocytes ratio), and (F) anti-NP antibody concentration. The letters in panel A, C, E, and F refer to monthly differences (significant levels from the Tukey's test). Note: *y*-axis of anti-NP antibody concentrations is minusLN-scaled.

**Figure 3:**
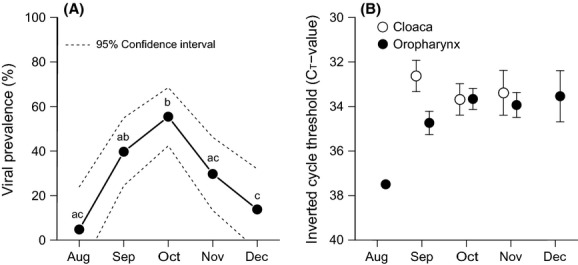
Monthly (A) viral prevalence (± 95% CI) and (B) degree of virus shedding (i.e., *C*_T_-value; mean ± SE) in cloacal and oropharyngeal samples. The *C*_T_-value is inversely proportional to the number of virus particles in a sample, with lower *C*_T_ values indicating a high amount of virus. The letters in panel A refer to the differences in viral prevalence between months (significant levels from the Tukey's test). Note inverted *y*-axis in panel B.

Body mass and immune status, except agglutination and Hp, varied over the autumn study period (Table2, Fig.2). Viral prevalence differed between months (GLM: *χ*^2^ = 38.92, *P *<* *0.001; Fig.3A), with no monthly difference in the degree of virus shedding from the cloaca and oropharynx (respectively, LM: *F*_2,40_ = 0.42, *P *=* *0.662 and *F*_4,86_ = 0.94, *P *=* *0.443; Fig.3B).

**Table 2:** Model output produced by the linear mixed models (LMMs) used to test the association between LPAIV infection and the six physiological variables in free-living mallards. The df is applicable for each model (*n *=* *287). Significant correlations are in bold

Variable	df	Body mass		Natural antibodies		Complement		Haptoglobin		H:L ratio		Anti-NP antibodies	
		*χ* ^2^	*P*-value	*χ* ^2^	*P*-value	*χ* ^2^	*P*-value	*χ* ^2^	*P*-value	*χ* ^2^	*P*-value	*χ* ^2^	*P*-value
Infection status	1	0.06	0.800	0.01	0.945	0.00	0.954	1.07	0.300	2.99	0.084	0.00	0.980
Age	1	12.49	**<0.001**	1.26	0.261	0.00	0.989	0.42	0.515	0.58	0.444	0.04	0.844
Sex	1	1.23	0.268	7.03	**0.008**	2.06	0.151	0.82	0.366	2.07	0.150	0.44	0.506
Migratory strategy	2	69.13	**<0.001**	6.15	**0.046**	1.61	0.447	8.10	**0.018**	4.43	0.109	4.04	0.132
Month	4	93.85	**<0.001**	3.19	0.526	15.12	**0.004**	4.42	0.352	21.94	**<0.001**	49.42	**<0.001**
Infection status × age	1	0.82	0.366	0.45	0.502	0.97	0.325	7.87	**0.005**	0.12	0.726	1.62	0.203
Infection status × sex	1	0.56	0.456	4.48	**0.034**	3.55	0.059	4.56	**0.032**	0.00	0.966	1.02	0.312
Infection status × migratory strategy	2	1.09	0.579	6.19	**0.045**	1.68	0.432	3.06	0.217	0.55	0.761	3.24	0.198
Infection status × month	4	6.26	0.180	12.22	**0.016**	46.19	**<0.001**	10.43	**0.034**	3.60	0.462	5.64	0.228
Bird size	1	89.95	**<0.001**										
Sample redness	1							20.33	**<0.001**				

### Body mass

**Figure 4:**
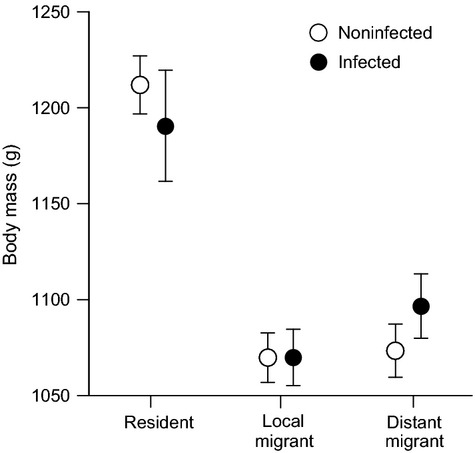
Body mass (mean ± upper/lower SE) of noninfected and LPAIV-infected residents, local migrants (i.e., coming from a short distance), and distant migrants (i.e., coming from a long distance).

All two-way interactions involving LPAIV infection were nonsignificant (Table2). Body mass, when corrected for bird size, did not differ between LPAIV-infected and noninfected birds (Table2). Juveniles had a lower body mass than adults (Table2). Local and distant migrants weighed less than residents (Tukey: both *P *<* *0.001; Table2, Fig.4). Sex was unimportant either as a main effect or in an interaction with infection status.

### Natural antibodies

**Figure 5:**
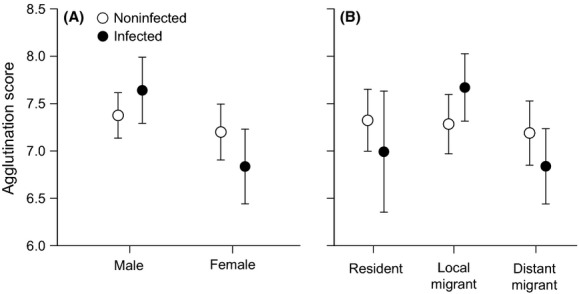
Agglutination scores (NAbs; mean ± SE) of noninfected and LPAIV-infected (A) males and females and (B) infected residents, local migrants, and distant migrants.

Three two-way interactions involving LPAIV infection were significant (Table2). Agglutination was higher in LPAIV-infected males compared to infected females (Fig.5A); agglutination was higher in infected local migrants compared to infected distant migrants (Fig.5B), and agglutination in a single infected bird in August was lower than in infected birds in the following months. Age class was unimportant either as a main effect or in an interaction with infection status.

### Complement

Only the two-way interaction between LPAIV infection and month was significant (Table2): an LPAIV-infected mallard in August had lower lysis than infected birds in the following months. Age class, sex, and migratory strategy were unimportant either as main effects or in interactions with infection status.

### Haptoglobin

**Figure 6:**
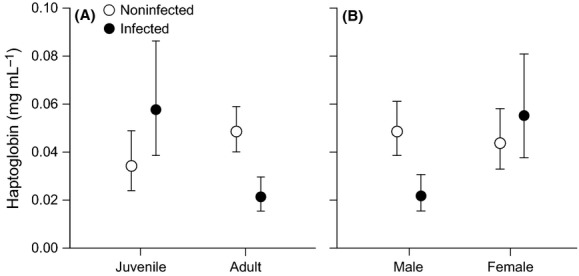
Haptoglobin concentrations (mean ± upper/lower SE) of noninfected and LPAIV-infected (A) juveniles and adults and (B) males and females.

Three two-way interactions involving LPAIV infection were significant (Table2). Hp concentrations were higher in LPAIV-infected juveniles compared to infected adults (Fig.6A), and Hp concentrations were higher in infected females compared to infected males (Fig.6B). An LPAIV-infected bird in August had higher Hp concentrations than the following months, whereas infected birds in September and November had lower Hp concentrations. Hp differed by migratory strategy: local migrants had higher Hp concentrations than residents (Tukey: *P *=* *0.014), with similar Hp concentrations as distant migrants (*P *=* *0.224).

### H:L ratio

All two-way interactions involving LPAIV infection were nonsignificant (Table2). H:L ratios did not differ between LPAIV-infected and noninfected birds (Table2). Age class, sex, and migratory strategy were unimportant either as main effects or in interactions with infection status.

### Anti-NP antibodies

All two-way interactions involving LPAIV infection were nonsignificant (Table2). Anti-NP antibody concentrations did not differ between LPAIV-infected and noninfected birds (Table2). As main effects and in interactions with infection status, age class, sex, and migratory strategy were unimportant.

### Virus shedding as an explanatory variable

We repeated all analyses after substituting infection status with virus shedding from the cloaca or virus shedding from the oropharynx. All two-way interactions involving virus shedding (regardless of location) were nonsignificant for all response variables (Table S2). Likewise, the main effect of virus shedding was never significant for any response variable (Table S2). Age class, sex, and migratory strategy were unimportant either as main effects or in interactions with virus shedding (regardless of location).

## Discussion

### LPAIV infection and body mass

During the autumn LPAIV infection peak, infected mallards did not differ in body mass from noninfected birds, when corrected for age, sex, and migratory strategy. Our results contradict the findings of Latorre-Margalef et al. (2009a), who found that during autumn migration, migratory mallards infected with LPAIV weighted less (by almost 20 g) than noninfected counterparts. Furthermore, while we found no relationships with virus shedding, Latorre-Margalef et al. (2009a) report that juveniles with a higher body mass shed less virus particles.

In contrast to the study of Latorre-Margalef et al. (2009a), the current analysis explicitly accounted for the role of migratory strategy. Mallards in the present study were assigned a migratory strategy using feather *δ*^2^H values and additional criteria (i.e., time of capture, recapture rate, and molt). By sampling mallards comprehensively throughout autumn (approximately every 5 days), we were able to accurately assess when birds were present at our study site, and whether they were in molt. Although we cannot fully exclude errors in migratory assignment, we are confident that using the criteria (causing one-third of the birds to be excluded from this study) and the *δ*^2^H values in feathers that we were able to determine the migratory strategy of mallards at the study site.

After assigning mallards to three migratory groups, we found an association between migratory strategy and body mass. Irrespective of LPAIV infection, local and distant migrants had a lower body mass than residents. However, if we excluded migratory strategy from the current body mass analysis, we, like Latorre-Margalef et al. (2009a), found an association between LPAIV infection and body mass (LMM: *χ*^2^ = 4.78, *P *=* *0.029): LPAIV-infected mallards were 31 g lighter than noninfected individuals. This result suggests that differences in body mass were likely not explained by LPAIV infection, but instead by migratory strategy. It is unknown whether including migratory strategy as a factor in the analysis of Latorre-Margalef et al. (2009a) would change their results. But given that study's location and timing, there was probably little variation in migratory strategy because most sampled individuals were probably migrants. Another potential explanation of the difference between the two studies is that migratory mallards in the previous study were sampled at a staging site during refueling, while in our study, birds were sampled on the wintering grounds. It is not unreasonable to think that LPAIV infection might have a greater impact in birds that are physically challenged by migration and are therefore faced with a trade-off between long-distance flight and immune investment (Altizer et al. 2011). Our results show the importance of controlling for migratory strategy when examining associations between LPAIV infection and body condition.

### LPAIV infection and immunological indices

There were no differences in any of the immunological variables between LPAIV-infected and noninfected mallards. Likewise, immune status was not associated with the degree of virus shedding from neither the cloaca nor the oropharynx. This lack of relationships is curious given the ostensible links between the immune indices and AIV. NAbs, which are predominantly IgM, play a transient role in the immune response to AIV by contributing antibodies early in the infection (3–5 days postinfection), and by fixation of complement (Magor 2011). Complement is thought to be involved in host defense of AIV; however, the extent of complement activation may depend on AIV subtype (O'Brien et al. 2011). Markers of acute phase responses (e.g., Hp) increase with LPAIV infection in chickens (Sylte and Suarez 2012; Dadras et al. 2014). Lymphocytes are essential in controlling LPAIV infection in birds (e.g., reduce viral shedding; Suarez and Schultz-Cherry 2000), whereas heterophils play important roles in the initial replication and dissemination of HPAIV (Pantin-Jackwood and Swayne 2009). LPAIV frequently produces heterophilic-to-lymphocytic rhinitis, sinusitis, tracheitis, and bronchitis in birds (Pantin-Jackwood and Swayne 2009). Anti-NP antibodies are generally produced when (semi-wild) naïve mallards are experimentally or naturally infected with LPAIV (Kida et al. 1980; Tolf et al. 2013). In sum, despite mechanistic links with AIV, the measured indices suggest that LPAIV does not trigger strong immune responses in free-living mallards, effectively recapitulating the conclusion of Magor (2011).

Our results contradict findings of experimental studies demonstrating upregulation of innate immune genes in AIV-infected domestic ducks (Barber et al. 2010; Vanderven et al. 2012). The lack of relationships between immunological indices and LPAIV infection in the mallards in our study could be associated with their infection history. Many individuals likely had been infected prior to sampling. As LPAIV infections are relatively short (i.e., up to a week; Latorre-Margalef et al. 2009a), the innate response might still be upregulated in birds that tested AIV-negative when sampled.

The stage of an identified LPAIV infection (start, middle, or tail of infection) and whether the infection was a bird's first or second (or other) time having LPAIV may influence a bird's immune response. Primary infections are predicted in early autumn and secondary infections in late autumn. To further investigate this point, we included the interaction between month and LPAIV infection in the analyses. Our results showed, albeit with a very low sample size in August, that early autumn infections were associated with lower levels of NAbs, complement, and Hp (only in September). This may indicate that birds that were infected with LPAIV for the first time (primary infection) had low antibody-mediated immunity (Whiteman et al. 2006; Parejo and Silva 2009). This could apply to all mallards sampled in August, because complement titers were, irrespective of LPAIV infection, lower compared to birds that had been sampled in late autumn. No associations were found between secondary infections and any of the immunological indices.

LPAIV-infected males had higher NAbs titers, but lower Hp concentrations than infected females. Sex differences in immune function may be attributed to corresponding differences in hormones, exposure to pathogens, or allocation of resources to the immune system due to differences in behavior and physiology (Møller et al. 1998). Similar reasons might also explain the higher levels of NAbs and Hp concentrations in, respectively, infected local migrants compared to infected distant migrants, and local migrants compared to residents. Flight reduces level of NAbs and Hp in some avian study systems, but not others (Matson et al. 2012b; Nebel et al. 2012). That LPAIV-infected juveniles had higher Hp concentrations than infected adults might be explained by the fact that juveniles were immunologically naïve. The antibody-mediated immune response in free-living birds deteriorates when individuals get older (Cichoń et al. 2003).

### Immunological indices compared with other studies

NAbs, complement, and Hp in the free-living mallards in our study were higher than values from captive adult mallards that were naïve to LPAIV infection (i.e., all birds were anti-NP antibody negative; Hoye 2012). Complement and Hp concentration were also higher in our study than values from young mallards in captivity, but NAbs titers of these birds were comparable to our results (Matson et al. 2005; Lee et al. 2012).

H:L ratios of free-living mallards in our study were comparable to H:L ratios found in captive, adult mallards (Fairbrother and Oloughlin 1990), but lower than H:L ratios in captive, young mallards (Yoder et al. 2006). The high H:L ratios in the young captives could reflect stress induced by captivity (Davis et al. 2008). In our study, mallards had higher H:L ratios during the LPAIV infection peak in October than at the start or end of the peak. This may indicate that in the months when LPAIV prevalence was highest, a larger proportion of birds was susceptible to LPAIV infection (Davis et al. 2008).

We measured anti-NP of AIV antibodies, which circulate in birds infected with any LPAIV subtype (Suarez and Schultz-Cherry 2000). Anti-NP antibody concentrations in our study were lower than in free-living and captive, adult mallards that were sampled in spring at our study site (van Dijk et al. 2014b). As the last major LPAIV infection peak was in autumn 2009 (van Dijk et al. 2014a), it is likely that mallards that were sampled 6 months later (spring 2010) have higher anti-NP antibody concentrations than those sampled a year later (autumn 2010). This could also explain that both LPAIV-infected and noninfected individuals in our study had low anti-NP antibody concentrations.

## Conclusion

Studying the physiological effects of LPAIV infection in waterfowl is important for generating better perspectives on their potential role as asymptomatic carriers of this virus. We studied associations between LPAIV infection and virus shedding, and six physiological variables in free-living mallards during the autumn infection peak on the birds' wintering grounds. The mid-autumn peak in viral prevalence found in our study is comparable to patterns from other studies of free-living mallards during the northern hemisphere autumn (Munster et al. 2007; Runstadler et al. 2007; Latorre-Margalef et al. 2014). We found no differences in body condition and only small differences in immune status that could potentially be attributed to LPAIV infection. The weak associations between LPAIV infection and mallards physiology highlight the potential for the species to function as an asymptomatic carrier of this virus. This possibility raises the intriguing questions about the coevolutionary history between mallards and LPAIV. Hosts and pathogens may coevolve if their relationship is close, and if strong selective pressures act on both the host and pathogen (Woolhouse et al. 2002). Factors such as susceptibility and virulence often depend on host–pathogen coevolutionary processes. For example, hosts may minimize virulence (i.e., mild or no disease effects) without minimizing pathogen fitness (Little et al. 2010). The extent of coevolution in the mallard-LPAIV system is unknown and requires further investigation. One promising route to a fuller understanding of the role of mallards in LPAIV infection dynamics may be looking for a gene-for-gene relationship (i.e., a single locus in the genome of both host and parasite; Woolhouse et al. 2002).

## Supporting Information

Table S1. Pairwise correlation coefficients between the six physiological variables. Table S2. Model output to test the association between the degree of virus shedding and the six physiological variables.
