# Supplementary material for: Minor differences in body condition and immune status between avian influenza virus-infected and noninfected mallards: a sign of coevolution?
Source: Ecol Evol. 2014 Dec 31;5(2):436–49. doi: 10.1002/ece3.1359 (PMC4314274; doi:10.1002/ece3.1359)
Supplement: Supplementary file 1 [file ece30005-0436-sd1.docx]

**Supporting Information**

**Table S1.** Pairwise correlation coefficients between body mass, agglutination score, lysis score, haptoglobin concentration, H:L ratio (heterophils to lymphocytes ratio), and anti-NP antibody concentration in free-living mallards.

|  |  |  |  |  |
| --- | --- | --- | --- | --- |
| Correlation | *r* | *t-*value | *P*-value | *r*^2^ |
| Body mass * Agglutination score | 0.04 | 0.692 | 0.489 | 0.00 |
| Body mass * Lysis score | -0.01 | -0.243 | 0.809 | 0.00 |
| Body mass * Haptoglobin | **-0.14** | **-2.422** | **0.016** | **0.02** |
| Body mass * H:L ratio | **-0.16** | **-2.762** | **0.006** | **0.02** |
| Body mass * Anti-NP antibodies | -0.02 | -0.339 | 0.735 | 0.00 |
| Agglutination score * Lysis score | **0.44** | **8.233** | **<0.001** | **0.19** |
| Agglutination score * Haptoglobin | **0.13** | **2.293** | **0.023** | **0.01** |
| Agglutination score * H:L ratio | 0.08 | 1.400 | 0.163 | 0.00 |
| Agglutination score * Anti-NP antibodies | 0.00 | -0.001 | 0.999 | 0.00 |
| Lysis score * Haptoglobin | 0.02 | 0.387 | 0.699 | 0.00 |
| Lysis score * H:L ratio | 0.08 | 1.306 | 0.193 | 0.00 |
| Lysis score * Anti-NP antibodies | 0.06 | 1.015 | 0.311 | 0.00 |
| Haptoglobin * H:L ratio | **0.14** | **2.396** | **0.017** | **0.02** |
| Haptoglobin * Anti-NP antibodies | 0.04 | 0.739 | 0.460 | 0.00 |
| H:L ratio * Anti-NP antibodies | 0.02 | 0.264 | 0.792 | 0.00 |

**Table S2**. Model output produced by the linear models (LMs) used to test the association between the degree of virus shedding (C_T_-value) and the six physiological variables in free-living mallards. (A) Cloaca samples, and (B) oropharyngeal samples. The *df* is applicable for each model (*n* = 287). Significant correlations are in bold.

| **A** |  |  |  |  |  |  |  |  |  |  |  |  |  |
| --- | --- | --- | --- | --- | --- | --- | --- | --- | --- | --- | --- | --- | --- |
|  |  |  |  |  |  |  |  |  |  |  |  |  |  |
|  |  | Body mass | | Natural antibodies | | Complement | | Haptoglobin | | H:L ratio | | Anti-NP antibodies | |
| Variable | *df* | *F*- value | *P*-value | *F*- value | *P*-value | *F*- value | *P*-value | *F*- value | *P*-value | *F*- value | *P*-value | *F*- value | *P*-value |
| C_T_-value | 1 | 0.03 | 0.860 | 1.82 | 0.187 | 3.92 | 0.057 | 0.83 | 0.369 | 0.18 | 0.678 | 0.01 | 0.943 |
| Age | 1 | 0.02 | 0.894 | 6.03 | **0.020** | 0.70 | 0.411 | 4.16 | 0.051 | 5.62 | **0.025** | 0.62 | 0.436 |
| Sex | 1 | 32.07 | **<0.001** | 3.94 | 0.057 | 0.31 | 0.581 | 5.84 | **0.022** | 0.61 | 0.442 | 3.06 | 0.091 |
| Migratory strategy | 2 | 9.22 | **<0.001** | 1.07 | 0.357 | 0.62 | 0.545 | 0.73 | 0.489 | 0.53 | 0.596 | 0.98 | 0.386 |
| Month | 2 | 4.95 | **0.014** | 0.80 | 0.460 | 1.22 | 0.311 | 2.39 | 0.110 | 3.62 | **0.040** | 0.96 | 0.394 |
| C_T_-value * age | 1 | 0.45 | 0.508 | 1.92 | 0.176 | 2.77 | 0.107 | 0.06 | 0.806 | 0.01 | 0.944 | 0.53 | 0.474 |
| C_T_-value * sex | 1 | 1.24 | 0.275 | 0.55 | 0.465 | 1.36 | 0.253 | 1.07 | 0.311 | 2.88 | 0.100 | 0.16 | 0.696 |
| C_T_-value * migratory strategy | 2 | 2.71 | 0.084 | 1.35 | 0.275 | 1.82 | 0.180 | 0.18 | 0.838 | 2.51 | 0.099 | 0.06 | 0.939 |
| C_T_-value * month | 2 | 1.04 | 0.366 | 0.32 | 0.730 | 1.18 | 0.321 | 1.51 | 0.237 | 0.06 | 0.946 | 1.10 | 0.347 |
| Bird size | 1 | 13.55 | **<0.001** |  |  |  |  |  |  |  |  |  |  |
| Sample redness | 1 |  |  |  |  |  |  | 6.34 | **0.018** |  |  |  |  |

| **B** |  |  |  |  |  |  |  |  |  |  |  |  |  |
| --- | --- | --- | --- | --- | --- | --- | --- | --- | --- | --- | --- | --- | --- |
|  |  |  |  |  |  |  |  |  |  |  |  |  |  |
|  |  | Body mass | | Natural antibodies | | Complement | | Haptoglobin | | H:L ratio | | Anti-NP antibodies | |
| Variable | *df* | *F*- value | *P*-value | *F*- value | *P*-value | *F*- value | *P*-value | *F*- value | *P*-value | *F*- value | *P*-value | *F*- value | *P*-value |
| C_T_-value | 1 | 0.86 | 0.358 | 1.76 | 0.188 | 0.29 | 0.589 | 0.00 | 0.989 | 0.45 | 0.506 | 0.01 | 0.904 |
| Age | 1 | 0.23 | 0.630 | 0.11 | 0.740 | 0.05 | 0.827 | 1.17 | 0.283 | 0.56 | 0.455 | 0.20 | 0.655 |
| Sex | 1 | 60.90 | **<0.001** | 7.52 | **0.008** | 10.73 | **0.002** | 5.84 | **0.018** | 0.26 | 0.614 | 1.59 | 0.211 |
| Migratory strategy | 2 | 3.55 | **0.034** | 6.01 | **0.004** | 1.81 | 0.170 | 3.91 | **0.024** | 0.59 | 0.559 | 1.47 | 0.236 |
| Month | 4 | 3.97 | **0.006** | 3.11 | **0.020** | 25.82 | **<0.001** | 2.26 | 0.071 | 3.02 | **0.023** | 4.08 | **0.005** |
| C_T_-value * age | 1 | 0.32 | 0.574 | 2.09 | 0.153 | 0.07 | 0.799 | 0.04 | 0.840 | 0.00 | 0.976 | 0.73 | 0.394 |
| C_T_-value * sex | 1 | 0.01 | 0.911 | 0.90 | 0.347 | 0.88 | 0.350 | 1.08 | 0.302 | 1.19 | 0.279 | 0.00 | 0.997 |
| C_T_-value * migratory strategy | 2 | 2.34 | 0.103 | 0.42 | 0.656 | 2.13 | 0.126 | 1.06 | 0.351 | 0.25 | 0.777 | 1.52 | 0.226 |
| C_T_-value * month | 3 | 0.74 | 0.531 | 0.42 | 0.738 | 0.59 | 0.622 | 0.14 | 0.934 | 2.31 | 0.083 | 0.27 | 0.849 |
| Bird size | 1 | 9.29 | **0.003** |  |  |  |  |  |  |  |  |  |  |
| Sample redness | 1 |  |  |  |  |  |  | 1.05 | 0.310 |  |  |  |  |
